# Gene expression signature of human neuropathic pain identified through transcriptome analysis

**DOI:** 10.3389/fgene.2023.1127167

**Published:** 2023-02-03

**Authors:** Ling Hu, Wei Yin, Yao Ma, Qiushi Zhang, Qingbang Xu

**Affiliations:** ^1^ Tianyou Hospital, Affiliated to Wuhan University of Science and Technology, Wuhan, China; ^2^ Department of Pain Medicine, Union Hospital, Tongji Medical College, Huazhong University of Science and Technology, Wuhan, China

**Keywords:** neuropathic pain, transcriptome, gene signature, diagnosis, therapeutic targets

## Abstract

**Introduction:** Neuropathic pain is a type of chronic pain that is characterized by ongoing discomfort and can be challenging to manage effectively. This study aimed to identify genes associated with neuropathic pain through transcriptome analysis in order to gain a better understanding of the mechanisms underlying this chronic, difficult-to-treat pain.

**Methods:** We conducted transcriptome analysis using a training datasetof 202 individuals, including patients with neuropathic pain and healthy controls.

**Results:** Our analysis identified five genes (GTF2H2, KLHL5, LRRC37A4P, PRR24, and MRPL23) that were significantly differentially expressed in the tissue of patients with neuropathic pain compared to controls. We constructed a neuropathic pain signature using these five genes and validated it using an independent dataset of 25 individuals. Receiver operating characteristic (ROC) curve analysis demonstrated that this signature had a high level of accuracy in differentiating between neuropathic pain patients and healthy controls, with an area under the curve (AUC) of 0.83 (95% CI 0.65–1).

**Discussion:** These findings suggest that these five genes may be potential therapeutic targets for neuropathic pain.

## Introduction

Neuropathic pain is a type of chronic pain that affects 7%–10% of the general population (Colloca et al., 2017). It is caused by damage or disease of the somatosensory system, which includes both peripheral fibers and central neurons (Baron et al., 2010). In humans, neuropathic pain results from sensitization of the peripheral and/or central nervous systems following an injury to these systems. Symptoms of neuropathic pain include hyperalgesia, allodynia, and spontaneous pain (Piao et al., 2018). Clinically, neuropathic pain is characterized by persistent pain or pain that shoots and grows in response to both noxious and non-noxious stimuli. There are many potential causes of neuropathic pain, including physical injuries, such as a nerve injury or amputation; medical conditions, such as diabetes, cancer, or multiple sclerosis; and certain medications or treatments, such as chemotherapy or surgery (Moisset et al., 2020; Noori et al., 2019). Other potential causes of neuropathic pain include infections, inflammation, and compression of the nerves. In some cases, the cause of neuropathic pain may be unknown (Amaniti et al., 2019).

The treatment of neuropathic pain often involves a combination of medications, physical therapy, and lifestyle changes. The specific treatment approach will depend on the underlying cause of the pain and the individual’s specific needs and circumstances.

Medications are a common treatment option for neuropathic pain. These may include over-the-counter pain relievers, such as acetaminophen or ibuprofen, or prescription medications, such as antidepressants, anticonvulsants, or opioids (O'Connor and Dworkin, 2009). These medications can help to reduce pain and improve function, but they may also have side effects and may not be effective for everyone. Physical therapy and other forms of rehabilitation can also be helpful in managing neuropathic pain. These may include exercises to improve strength and flexibility, as well as techniques to reduce stress and improve overall physical and mental health. Lifestyle changes, such as getting regular exercise, eating a healthy diet, and managing stress, can also be beneficial in managing neuropathic pain (Akyuz and Kenis, 2014). It may also be helpful to work with a pain psychologist or other mental health professional to develop coping strategies and manage the emotional impact of living with chronic pain (Wallace, 2007). It is important for individuals experiencing neuropathic pain to work with their healthcare team to develop a treatment plan that is tailored to their specific needs and circumstances.

Our study aimed to investigate the molecular mechanisms of neuropathic pain and identify potential therapeutic targets by analyzing the genome-wide mRNA profiles in order to establish a diagnostic signature for neuropathic pain. By gaining a deeper understanding of the pathogenesis of neuropathic pain through the identification of this signature, we hope to improve our ability to effectively treat this condition, which currently lacks clear molecular understanding and effective treatment options.

## Methods

### Gene expression analysis

We used the R package MetaIntegrator to integrate the results of our discovery study, which aimed to identify genes that are differentially expressed between controls and patients with chronic pain (Haynes et al., 2017). We calculated Hedges’ adjusted g values for each gene and applied Benjamini–Hochberg false discovery rate (FDR) correction to the Student’s *t*-test values to adjust for gene effect sizes. This allowed us to identify genes that may be involved in the development of chronic pain.

### Bioinformatical analysis

We obtained RNA-seq raw data for our discovery and validation datasets from the Gene Expression Omnibus (GEO) (Dawes et al., 2014; Parisien et al., 2022). The discovery dataset (GSE177034) consisted of 98 participants with neuropathic pain who had blood samples collected at the beginning and end of the study. We compared peripheral blood gene expression in patients with neuropathic pain whose low back pain (LBP) resolved after 3 months with those whose LBP persisted. The second discovery dataset (GSE54413) consisted of six skin samples from genome-wide transcriptional profiling.

### Data pre-processing and statistics analysis

We used the nf-core RNA-seq pipeline version 3.6 (https://nf-co.re/rnaseq) to quantify RNA-seq expression in our discovery and validation datasets. We calculated the statistical power for the meta-analysis of gene expression between controls and chronic pain patients in both datasets, with a focus on detecting effect sizes greater than 0.4 at a *p*-value of less than 0.1. We defined significant enrichment as a *p*-value of less than 0.05. All statistical analyses were performed using the R software (Version 4.2.0).

### Independent validation cohort

Patients with low back pain (LBP) were classified according to published criteria (Carragee and Hannibal, 2004),Briefly, the diagnosis of low back pain (LBP) is a complex process that involves a comprehensive evaluation of the patient’s medical history, symptoms, physical examination, and imaging studies. The standard for LBP diagnosis is based on the criteria established by the International Association for the Study of Pain (IASP). It states that LBP is a “pain perceived in the area below the costal margin and above the inferior gluteal folds, with or without leg pain.” Additionally, the patient must have had the symptoms for at least 3 months. However, other definitions of LBP can be based on specific subtype of low back pain, like mechanical low back pain, or specific population, like low back pain in older adults. Diagnosis of LBP is usually made through clinical examination by a healthcare provider and it is important to note that LBP can have many potential causes, such as muscle strain, disk injury, or spinal stenosis, and that the underlying cause of LBP should be identified if possible. Differential diagnosis should also be made for red flags like malignant tumors, infections, fractures or osteoporotic vertebral collapse which might be related with severe pain. In-depth clinical information can be found in the Supplementary Table S1, which includes patient demographics, disease characteristics and other relevant data. To ensure balanced clotting, blood was collected in an EDTA-containing vacutainer tube. The plasma was then transferred to a clean tube and centrifuged again at 3,000 g for 10 min. One milliliter of plasma was transferred to a clean tube after the centrifugation step and stored at −80°C for further processing.

### RT-PCR assay

The RT-PCR assay was performed to quantify the expression levels of specific transcripts in a sample. Total RNA was first isolated from blood sample and reverse transcribed into cDNA using the High Capacity Reverse Transcription kit (ABI). The qPCR methodology were used according to the protocols described in published articles (Pfaffl, 2001). The details of the primers can be found in the Supplementary Table S2.

## Results

### Identification of the neuropathic pain signature using transcriptome analysis

**FIGURE 1:**
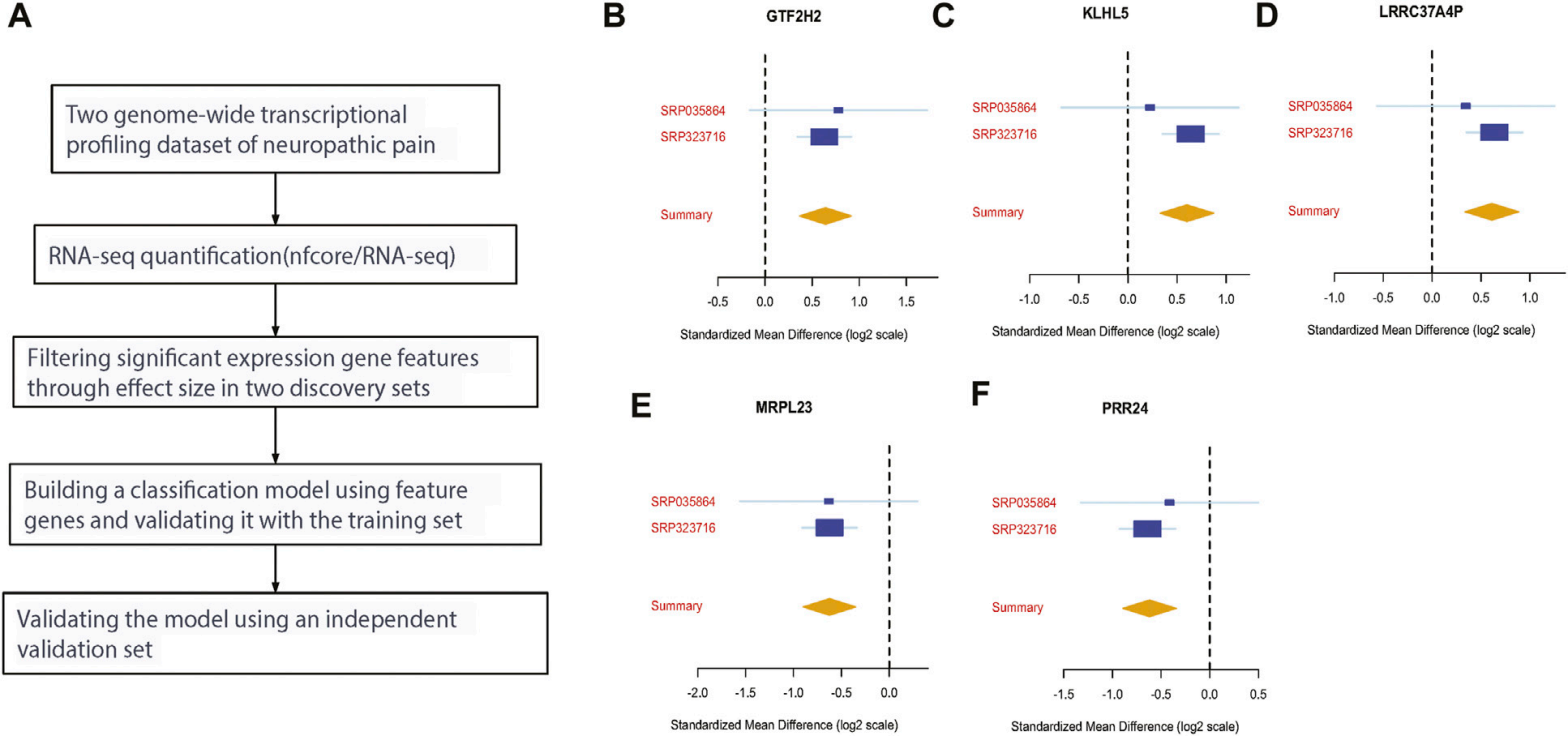
Discovery of five differentially expressed genes in neuropathic pain. **(A)** An analysis workflow was used to identify and validate three differentially expressed genes across training and validation datasets; **(B–F)** the effect sizes of four upregulated genes (GTF2H2, KLHL5, LRRC37A4P, and MRPL23) and two downregulated genes (MRPL23 and PRR24) were analyzed.

Figure 1A shows the workflow for our study. We used publicly available datasets from GEO to identify key biomarkers and drug targets for neuropathic pain. We compared the mRNA expression profiles in the neuropathic pain and control groups using RNA-seq. To identify differentially expressed genes, we used the MetaIntegrator framework to select a set of DE genes from the discovery and validation datasets. We identified 14 significantly DE genes (12 upregulated and two downregulated). After applying the filtering criteria described in the Methods section, we chose six DE genes as final biomarkers (Figures 1B–F).

### Validation of the neuropathic pain signature by independent validation dataset

**FIGURE 2:**
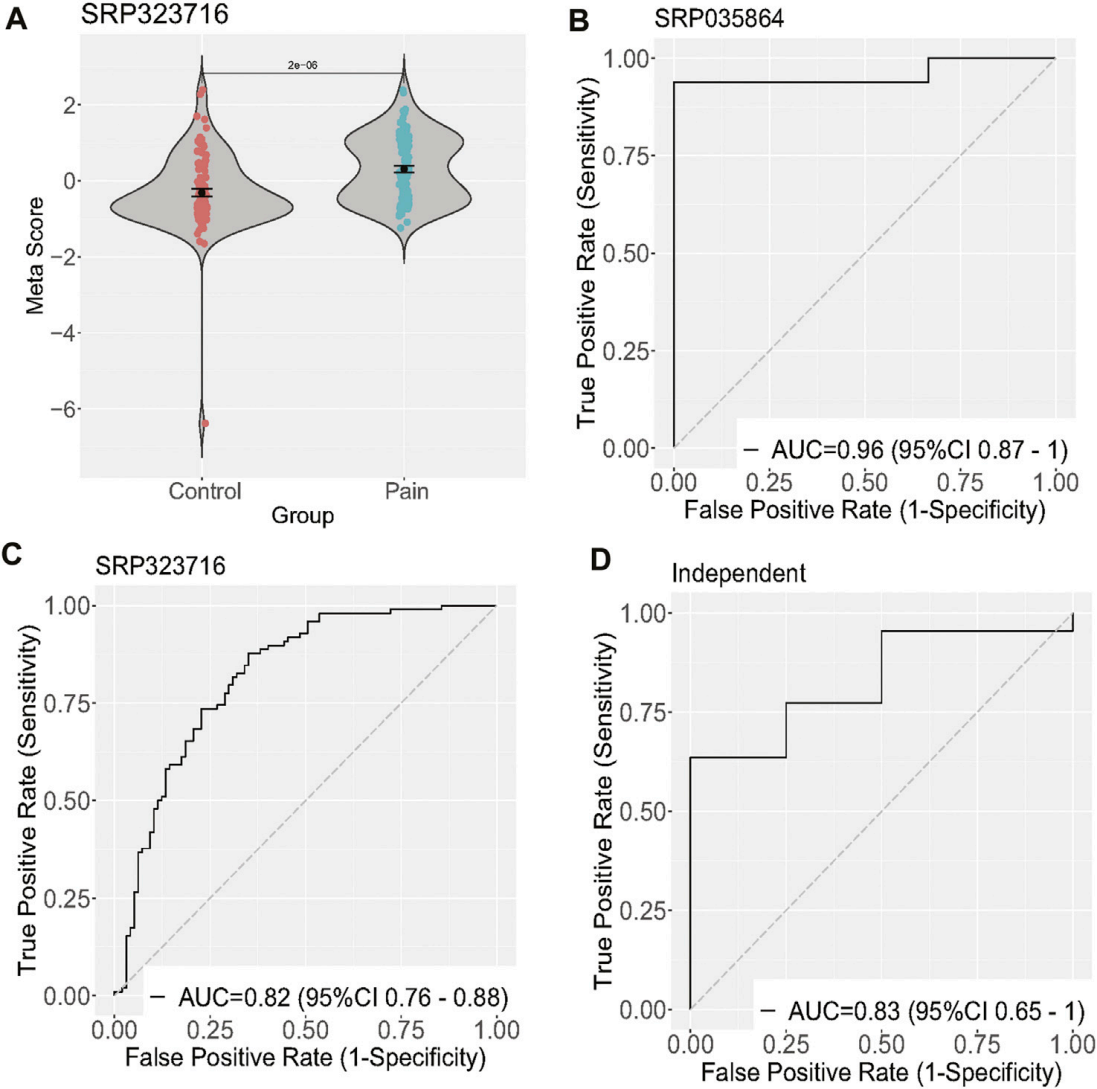
Validation of five differentially expressed genes of neuropathic pain. **(A)** Diagram illustrating the meta-score of one training dataset; **(B–D)** ROC curves of patients with neuropathic pain versus controls in two training and one validation dataset.

To validate the differentially expressed genes (GTF2H2, KLHL5, LRRC37A4P, PRR24, and MRPL23), we calculated a neuropathic pain score for each sample by taking the mean expression of the over-expressed genes and subtracting the mean expression of the under-expressed gene (Figures 2A, B). In the discovery dataset, the five-gene neuropathic pain scores were able to differentiate neuropathic pain from the control group with an area under the curve (AUC) of 0.96 (95% CI 0.87–1) and 0.82 (95% CI 0.76–0.88). We then validated this signature in the validation dataset (26 samples), which had significant clinical heterogeneity, including differences in sample type, age, race, and treatment criteria. Despite these differences, the scores accurately identified neuropathic pain patients with an AUC of 0.83 (95% CI 0.65–1) (Figures 2C, D).

## Discussion

Treatment of neuropathic pain is challenging, and most existing therapies are ineffective or have harmful side effects (Rosenberger et al., 2020). Therefore, understanding the underlying mechanisms of neuropathic pain is critical for developing new therapeutic strategies to manage and control this condition. In this study, we analyzed the transcriptome of patients with neuropathic pain using blood samples collected at the beginning and end of the study. We identified six genes that were differentially expressed between the control and neuropathic pain groups. A specific signature of GTF2H2, KLHL5, LRRC37A4P, PRR24, and MRPL23 was able to differentiate neuropathic pain from normal. We also validated this signature using an independent validation set. Interestingly, the expression of MRPL23 and PRR24 was decreased in the neuropathic pain group. Mitochondrial ribosomal protein L23 (MRPL23) is encoded by nuclear genes and plays a role in protein synthesis within mitochondria (Li et al., 2022). Two diseases, Familial Wilms Tumor 2 and Wilms Tumor 2, have been associated with MRPL23. To the best of our knowledge, this is the first time MRPL23 has been linked to neuropathic pain in humans (Matsunaga, 1981). PRR24 (proline rich 24) is a protein-coding gene located on chromosome 14 in humans. It belongs to the proline-rich protein family, which is characterized by the presence of multiple proline residues. PRR24 is a ubiquitously expressed protein that is involved in various cellular processes, including cell adhesion and migration (Pukrittayakamee et al., 2000). The general transcription factor IIH subunit 2 (GTF2H2) is part of a 500 kb inverted duplication on chromosome 5q13. Duplicated regions often include at least four genes and repetitive elements, which tend to undergo rearrangement and deletion (Karasu et al., 2022).

The results of our study provide insights into the molecular mechanisms of neuropathic pain and suggest potential therapeutic targets. The identification of MRPL23 as a differentially expressed gene in neuropathic pain is particularly noteworthy, as it has not previously been linked to this condition. Further research is needed to understand the specific role of MRPL23 in neuropathic pain and to determine whether it could be a potential target for therapy. The other differentially expressed genes identified in our study, also warrant further investigation to determine their potential roles in neuropathic pain and their potential as therapeutic targets. Recent studies have identified KLHL5 and LRRC37A4P as potential genetic markers for certain diseases. KLHL5, also known as Kelch-like protein 5, has been shown to be involved in the development of cancer, particularly in the regulation of cell proliferation and apoptosis (Schleifer et al., 2018). On the other hand, LRRC37A4P, also known as leucine-rich repeat-containing protein 37A4 pseudogene, has been associated with an increased risk of cardiovascular disease (Su et al., 2021). Both genes have been identified as key players in the regulation of cellular processes, making them potential therapeutic targets for the treatment of disease. However, further research is needed to fully understand the role of these genes in disease development and progression.

In addition to identifying differentially expressed genes, our study also demonstrated the utility of using a gene expression signature to diagnose neuropathic pain. The neuropathic pain score that we developed was able to accurately differentiate neuropathic pain from normal in both the discovery and validation datasets. This signature could potentially be used as a diagnostic tool in clinical practice to help identify patients with neuropathic pain and guide treatment decisions. Overall, our study adds to the growing body of knowledge on the molecular mechanisms of neuropathic pain and provides new insights into potential therapeutic targets and diagnostic approaches. Further research is needed to fully understand the pathogenesis of neuropathic pain and to develop more effective treatments for this debilitating condition.

## Data Availability

The data presented in the study are deposited in the GEO repository (https://www.ncbi.nlm.nih.gov/geo/), accession number GSE177034.
