# Supplementary material for: Gene expression signature of human neuropathic pain identified through transcriptome analysis
Source: Front Genet. 2023 Feb 3;14:1127167. doi: 10.3389/fgene.2023.1127167 (PMC9936241; doi:10.3389/fgene.2023.1127167)
Supplement: Supplementary file 1 [file Table1.DOCX]

**Supplementary**

Supplementary table 1: Demographic and Clinical Characteristics of Patients with Low Back Pain and controls (n=25)

| Patient id | Age | Gender | Duration of LBP (months) | Pain intensity (VAS) | Type of LBP | Comorbidities | Medications |
| --- | --- | --- | --- | --- | --- | --- | --- |
| P1 | 44 | Male | 21 | 6 | Mechanical | Hypertension | Acetaminophen |
| P2 | 32 | Female | 12 | 7 | Radicular | None | Naproxen |
| P3 | 72 | Male | 33 | 8 | Non-Specific | Diabetes | Diclofenac |
| P4 | 41 | Female | 18 | 5 | Mechanical | Arthritis | Ibuprofen |
| P5 | 33 | Male | 6 | 6 | Non-Specific | None | Acetaminophen |
| P6 | 34 | Female | 24 | 8 | Radicular | Hypertension | Naproxen |
| P7 | 49 | Female | 44 | 6 | Mechanical | Arthritis | Diclofenac |
| P8 | 45 | Male | 48 | 7 | Non-Specific | None | Ibuprofen |
| P9 | 75 | Female | 42 | 5 | Mechanical | Diabetes | Acetaminophen |
| P10 | 35 | Male | 36 | 6 | Non-Specific | Hypertension | Naproxen |
| P11 | 66 | Female | 15 | 8 | Radicular | Arthritis | Diclofenac |
| P12 | 71 | Male | 7 | 5 | Mechanical | None | Ibuprofen |
| P13 | 60 | Male | 0 | 0 | None | Diabetes | None |
| P14 | 50 | Female | 0 | 0 | None | None | None |
| P15 | 55 | Female | 0 | 0 | None | None | None |
| P16 | 71 | Male | 0 | 0 | None | Diabetes | None |
| P17 | 61 | Female | 0 | 0 | None | None | None |
| P18 | 30 | Male | 0 | 0 | None | None | None |
| P19 | 64 | Female | 0 | 0 | None | Arthritis | None |
| P20 | 69 | Male | 0 | 0 | None | Arthritis | None |
| P21 | 42 | Female | 0 | 0 | None | None | None |
| P22 | 39 | Female | 0 | 0 | None | None | None |
| P23 | 72 | Male | 0 | 0 | None | Hypertension | None |
| P24 | 55 | Female | 0 | 0 | None | Hypertension | None |
| P25 | 48 | Female | 0 | 0 | None | None | None |

Supplementary Table 2: Primer Sequences of Genes GTF2H2, KLHL5, LRRC37A4P, PRR24, and MRPL23 for qPCR analysis

| ID | gene | Primer (5'- 3') |
| --- | --- | --- |
| 1 | ACTB | CGTGGATGGACTCCACGAC |
| 2 | ACTB | GTGGTGGTGAGGGAGCAG |
| 3 | GTF2H2 | GAAGTTCGCGTTTGCACTGT |
| 4 | GTF2H2 | TGGTGTGCTGAGGAAATCCC |
| 5 | KLHL5 | AGAAAAAGGGGGTGGTGTTCT |
| 6 | KLHL5 | CTGCGATCACCAGCGACTAA |
| 7 | LRRC37A4P | TGCTCTTCACGAAGGGAGAG |
| 8 | LRRC37A4P | AGCGGCCGTCTGAATGAAAA |
| 9 | PRR24 | GGAACTAGAGTGTGGGAGCC |
| 10 | PRR24 | GCTGTCGGACTGCTCTACTC |
| 11 | MRPL23 | GGCGCGGAATGTGGTGTA |
| 12 | MRPL23 | GTAGGCGACCTTGTAGTCCG |
